# Acetic acid treatment in *S. cerevisiae* creates significant energy deficiency and nutrient starvation that is dependent on the activity of the mitochondrial transcriptional complex Hap2-3-4-5

**DOI:** 10.3389/fonc.2012.00118

**Published:** 2012-09-21

**Authors:** Ana Kitanovic, Felix Bonowski, Florian Heigwer, Peter Ruoff, Igor Kitanovic, Christin Ungewiss, Stefan Wölfl

**Affiliations:** ^1^Institute for Pharmacy and Molecular Biotechnology, Heidelberg UniversityHeidelberg, Germany; ^2^Faculty of Science and Technology, Centre for Organelle Research, University of StavangerStavanger, Norway

**Keywords:** growth dynamic, response surface modeling, automated assay, acetic acid, Hap4p, intracellular pH, metabolic control

## Abstract

Metabolic pathways play an indispensable role in supplying cellular systems with energy and molecular building blocks for growth, maintenance and repair and are tightly linked with lifespan and systems stability of cells. For optimal growth and survival cells rapidly adopt to environmental changes. Accumulation of acetic acid in stationary phase budding yeast cultures is considered to be a primary mechanism of chronological aging and induction of apoptosis in yeast, which has prompted us to investigate the dependence of acetic acid toxicity on extracellular conditions in a systematic manner. Using an automated computer controlled assay system, we investigated and model the dynamic interconnection of biomass yield- and growth rate-dependence on extracellular glucose concentration, pH conditions and acetic acid concentration. Our results show that toxic concentrations of acetic acid inhibit glucose consumption and reduce ethanol production. In absence of carbohydrates uptake, cells initiate synthesis of storage carbohydrates, trehalose and glycogen, and upregulate gluconeogenesis. Accumulation of trehalose and glycogen, and induction of gluconeogenesis depends on mitochondrial activity, investigated by depletion of the Hap2-3-4-5 complex. Analyzing the activity of glycolytic enzymes, glyceraldehyde-3-phosphate dehydrogenase (GAPDH), pyruvate kinase (PYK), and glucose-6-phosphate dehydrogenase (G6PDH) we found that while high acetic acid concentration increased their activity, lower acetic acids concentrations significantly inhibited these enzymes. With this study we determined growth and functional adjustment of metabolism to acetic acid accumulation in a complex range of extracellular conditions. Our results show that substantial acidification of the intracellular environment, resulting from accumulation of dissociated acetic acid in the cytosol, is required for acetic acid toxicity, which creates a state of energy deficiency and nutrient starvation.

## Introduction

Basic metabolic pathways provide energy and molecular building blocks required for growth, maintenance and repair. Changes in the metabolome correlate with declining functions with age. Network analysis of metabolism can help to determine how failures in metabolic control, required to maintain stability and homeostasis within living systems, can lead to senescence and aging.

Acetic acid is a normal end product of alcoholic fermentation in *S. cerevisiae* that cannot be metabolized by glucose-repressed yeast cells. In undissociated form acetic acid is freely membrane permeable and enters the cell by simple diffusion. At higher extracellular pH acetic acid will dissociate to the acetate anion, a form that is relatively membrane-impairment. Acetic acid was also shown to induce apoptosis in yeast cells involving release of cytochrom c in a mitochondria dependent apoptotic pathway (Ludovico et al., 2002; Pereira et al., 2007). Almeida et al. (2009) presented that induction of apoptosis triggered by acetic acid is accompanied by severe amino acids starvation and activation of the TOR signaling pathway. Acetic acid was also identified as a cell-extrinsic mediator of cell death during chronological aging in *S. cerevisiae* (Burtner et al., 2009).

These observations suggest that the cellular response to acetic acid and the induction of apoptosis could strongly depend on specific combinations of extracellular conditions, like medium pH or glucose availability. To investigate how various combinations of extracellular factors and metabolic activity modulate the biological response of yeast cells to acetic acid treatment, we performed a large scale study using a computer-controlled robot-system together with a mathematical algorithm for experimental planning to appropriately cover the full space of multiple culture/medium parameters by automated variation of experimental conditions (Bonowski et al., 2010). Experiments on the robot systems are controlled by R scripts using object-oriented descriptions of experimental parameters. Thus, providing only a small set of defined stock solutions, yeast growth is automatically analyzed in a multidimensional parameter space. By covering the whole range of all possible combinations, we try to avoid a bias in experimental conditions that could influence the experimental results (Kovárová-Kovar and Egli, 1998; Narendranath and Power, 2005). The resulting datasets of growth kinetics depending on the variable parameters are visualized using multidimensional regression methods and response surface modeling that facilitate the determination of the optimum values for the factors under investigation. Using this automated procedure we provide a detailed model for the dynamic dependence of biomass yield and growth rate on extracellular glucose concentration, pH conditions and acetic acid concentration. Further investigation revealed that accumulation of acetic acid in the cytosol results in inhibition of the respiratory chain and ceased uptake of carbohydrates creating significant energy deficiency and nutrient starvation. Acetic acid also directly influenced the activity of key metabolic enzymes, glyceraldehyde-3-phosphate dehydrogenase (GAPDH), pyruvate kinase (PYK), and glucose-6-phosphate dehydrogenase (G6PDH).

## Materials and methods

### Strains and culturing conditions

The yeast strains used in this study are: FF 18984 (*MATa leu2-3,112 ura3-52, lys2-1, his7-1*) and Δ*hap4* (*MATa leu2-3,112 ura3-52, lys2-1, his7-1;hap4::KanMX4)*. Yeast cells were grown in rich (YPD) medium containing 10 g l^−1^ Yeast Extract, 20 g l^−1^ Bacto peptone, and different glucose concentrations (as indicated; 20, 10, 5 g, or 2.5 g l^−1^). Cell were grown over night at 30° with agitation, collected by centrifugation at 3500 rpm for 5 min, suspended in water and used as a cell stock for the assays.

### Growth dynamic experimental setup

Experiments were carried out by a Tecan Genesis RSP 150 robot and a Tecan Ultra II plate reader. The system is controlled by a set of R packages developed in our lab that provide a general experimental framework for fluid mixture based experiments. The program packages enable automated variation of experimental conditions by generating tables of concentrations of each assay component. Pipetting volumes are calculated from these concentrations and all pipetting and measurement steps are executed automatically. The robot pipettes all components into 96 U-shaped well plates and growth is automatically analyzed in a plate reader controlled by a modified version of the XFluor Excel macros. The protocols allow fully automated configuration, execution and export of measurement data without user-interactions [for more details see Bonowski et al. (2010)].

Medium stock solutions were defined as fixed components (4xYP-yeast extract/peptone/dextrose medium (YPD) and variable components (glucose, pH, and acetic acid). The parameter space for the variable medium components was specified in terms of concentrations and pH values and a *space-filling design* was used to cover it with measurement points, 48 conditions for each strain. The pH was controlled by combination of two buffers adjusted to different pH values (50 mM citrate/phosphate buffer). A spline fit method was applied to convert between buffer fractions and pH values. Medium components were pipetted first by multi-pipetting mode. Yeast cells in water were added to a final concentration of 0.2 OD_600_ and growth kinetics were measured at 620 nm for 114 cycles at an interval of 10 min. Reader thermostat was adjusted to 30°C.

### Quantitative assessment of glucose, glycogen, and trehalose content

In order to quantify glucose consumption and glycogen and trehalose accumulation, culture supernatant and cell pellet were collected at indicated time points. The procedure was performed as described previously (Parrou and Francois, 1997). Briefly, the cell pellet (collected from 20 OD_600_ units of culture) was suspended in 250 μl 0.25 M Na_2_CO_3_ and heated at 95°C for 4 h with occasional stirring. The suspension was adjusted to pH 5.2 with 150 μl 1 M acetic acid and 600 μl 0.2 M sodium acetate buffer, pH 5.2. Half of this mixture was incubated overnight at 57°C with continuous shaking on a rotary shaker in the presence of 100 μg of α-amyloglucosidase from *Aspergillus niger* (Sigma). The second half of the mixture was incubated overnight at 37°C in the presence of 3 mU trehalase (Sigma). The glucose released from glycogen and trehalose digestion as well the glucose content in the medium were determined with the glucose oxidase/peroxidase method (Cramp, 1967).

### Monitoring of oxygen consumption

Oxygen consumption was monitored in OxoPlate® (PreSens; Germany) covered with a breathable membrane (Diversified Biotech, USA) in 150 μl volumes containing the indicated medium conditions and 0.1 OD_600_ units of cells. The plates were prepared using our computer-controlled automated experimental design. The signal of the oxygen fluorescence sensor and the optical density of the culture at 600 nm were measured continuously during indicated times with intervals of 10 min. The calibration of the fluorescence reader was performed using a two-point calibration curve with oxygen-free water (80 mM Na_2_SO_3_) and air-saturated water. Partial pressure of oxygen was calculated from the calibration curve.

### Preparation of cell-free extracts and enzyme assays

All procedures were carried out at 0–4°C. Crude extracts were prepared from 20 OD_600_ units of cells with 1 g glass beads (0.4–0.5 mm diameter) in 0.5 ml 20 mM Hepes, pH 7.1, 100 mM KCl, 5 mM MgCl2, 1 mM EDTA, and 1 mM DTT. Samples were vortexed (3 × 5 min with cooling on ice in between) in Mixer Mill MM 300 (Retsch). After centrifugation at 16,000 g for 15 min at 4°C, the supernatants were immediately used for enzymatic assays. Protein content was determined by the method of Bradford (1976). All chemicals and enzymes for enzymatic assays were purchased from Sigma.

Glyceraldehyde-3-phosphate dehydrogenase (GAPDH; EC 1.2.1.12), pyruvate kinase (PYK; EC 2.7.1.40), and glucose-6-phosphate dehydrogenase (G6PDH; EC 1.1.1.49) activity were measured at 30°C by measuring NADH consumption or NADPH production, using a spectrophotometric assays as described earlier (Kitanovic et al., 2009).

Fructose 1,6 bisphosphatase (FBP1; EC 3.1.3.11) activity was measured at 30°C by coupling reactions of FBP1, phosphoglucoisomerase and G6PDH and measuring NADPH production, using a spectrophotometric assay. The reaction was made in 0.15 ml containing 100 mM Imidazole, pH 7.1, 100 mM KCl, 5 mM MgSO_4_, 5 mM EDTA, 0.7 mM NADP^+^, 3.4 mM glucose-6-phosphate, and 10 units of phosphoglucoisomerase and G6PDH (EC 5.3.1.9 and EC 1.1.1.49, respectively). The reaction was initiated by the addition of crude extract and increase of absorbance at 340 nm was monitored.

Malate dehydrogenase (MDH; EC 1.1.1.37) activity was measured at 30°C by monitoring NADH consumption, using a spectrophotometric assay. The reaction was made in 0.15 ml containing 50 mM Tris/MES, pH 7.4, 200 mM KCl, 10 mM MgCl_2_, 0.625 mM NADH, and 0.5 mM oxaloacetate. The reaction was initiated by the addition of crude extract and decrease of absorbance at 340 nm was monitored.

Isocitrate dehydrogenase (IDH; EC 1.1.1.41) activity was measured at 30°C by monitoring NADH production, using a spectrophotometric assay. The reaction was made in 0.15 ml containing 50 mM Tris/MES, pH 7.5, 100 mM KCl, 12 mM MgCl_2_, 0.625 mM NADH, and 8 mM isocitrate. The reaction was initiated by the addition of crude extract and decrease of absorbance at 340 nm was monitored.

### Monitoring of intracellular pH

For cytosolic expression of ratiometric pHluorin we used pHluorin in an expression plasmid with a strong constitutive ADH1 promoter, kindly provided by Tobias Dick (Heidelberg) (Braun et al., 2010). For estimating the calibration curve, the cells were resuspended in a series of 50 mM citrate-phosphate calibration buffers of defined pH, 5.0, 5.5, 6.0, 6.5, 7.0, 7.5, and 8.0 (supplements). Cells containing pHluorin reporter plasmid were grown in 260 μl low fluorescence minimal F1 medium (Kitanovic and Wölfl, 2006) at 30°C in Tecan Ultra microplate reader (Tecan). Kinetic parameters were measured at 390/510 and 480/510 nm Ex/Em. According to the calibration curve, we then calculated the internal pH values (pHin) from fluorescence ratios measured in the experimental part. Samples were measured in triplicates; the data shown represents one of three independent experiments.

### Data analysis

#### Multivariate response surface modeling

The large multidimensional datasets generated by our experimental framework require new approaches of explorative data-analysis. To visualize the response of yeast to changes in medium composition, we use 2D slices of multivariate response surface models (RSMs).

Classical RSMs based on low order polynomials have a long and successful history as a tool for optimizing biotechnological processes (Popa et al., 2007; Fereidouni et al., 2009; Singh et al., 2009). The main advantages of these models are that they are mathematically simple, easy to optimize and provide a method to improve processes without having to carry out a large number of measurements. Unfortunately, fitting a fixed functional form to a dataset introduces a massive bias and is unlikely to yield an accurate description of a complex non-linear system (Jones, 2001), making them unsuitable as an explorative tool.

Non-parametric and semi-parametric regression techniques like Gaussian Random Process Regression (GRPR) can help to avoid these shortcomings (Cressie, 1993). The assumptions behind GRPR are much more general than those behind classical RSMs, giving them much more flexibility to fit the data in a less biased fashion. In this work, we show how the combination of automated experimenting and visualization of datasets using GRPR can help to get an intuitive understanding of the combined quantitative influence of multiple factors on the growth dynamic of yeast cultures.

Yeast growth in a liquid batch culture was expressed as exponential rate constant μ(t) where:

(1) dOD(t)/dt=μ(t) · OD(t)

In order to define growth kinetic over time a piecewise linear regression of log (OD) was determined:

(2) $$\begin{array}{l} \text{OD}(\text{t})=\text{OD}({\text{t}}_{\text{0}})\;\cdot {\text{ e}}^{\mu (\text{t})*\text{t}}\\ \;\Rightarrow \text{ln}(\text{OD}(\text{t}))=\text{ln}(\text{OD}({\text{t}}_{\text{0}}))\text{ + t }\cdot \;\mu (\text{t}) \end{array}$$

We developed an extension to the GRPR implemented in the *fields* R package for visualizing slices of multidimensional datasets and obtaining non-parametric surrogate models of experimental systems. The *fields* implementation uses generalized cross validation to obtain an estimation of the noise-level of the data and find optimal smoothing parameter (Marcotte, 1995). Our implementation also performs an optimization of the length-scale parameter of the covariance function in all dimensions of the model using the Nelder-Mead method as implemented in the R function *optim* with cross-validation error of *fields* as the objective function, and allows scaling of the axes with arbitrary functions that reflect *a-priori* assumptions about the sensitivity of the system to parameter changes in different regions of the parameter space. Certain axes are switched to logarithmic scaling, which is useful for many biochemical systems that show a large variability at low concentrations (Bonowski et al., 2010).

GRPR has been used extensively in the field of geostatistics, where it is commonly called Kriging (Cressie, 1993). A Gaussian process is a collection of random variables, any finite number of which have a joint Gaussian distribution (Rasmussen and Williams, 2006). In our setting, each observation at a point *x*_*i*_ in the parameter space corresponds to one random variable of the Gaussian process. In GRPR, one assumes that the covariance of different observations is a function *k*(x_*i*_, x_*j*_) of their locations in the parameter space. The joint distribution *f(X)* of observations at a set *X* of parameter combinations is given by

(3) f(X)~N(μ, ∑ = K(X))

where *N* is a multivariate normal distribution and the entries *K*_*ij*_ = *k*(*x*_*i*_, *x*_*j*_) of the covariance matrix are given by the covariance function.

Predictions *f*(X^*^) for unknown parameter combinations X^*^ can be derived from the joint distribution with observations from measurements at locations *X* as described in Cressie (1993). The choice of a covariance function *k*(*x*_*i*_, *x*_*j*_) provides a way to influence on the properties of the resulting RSM.

Our models are based on a stationary covariance function of the “Gaussian” type that is one of the standard choices for a smooth covariance function (Cressie, 1993):

(4) $$k(x_{i},\;x_{j})=\sigma_{f}^{2}\exp\left(-\frac{1}{2l^{2}}{\left(x_{i}-x_{j}\right)}^{2}\right)+\sigma_{n}^{2}\delta_{ij}$$

In this formula, σ_*f*_ describes the strength of correlation between measurements with similar parameters, *l* describes the range of the correlation, σ_*n*_ describes the measurement noise, and δ_*ij*_ is 1 if *i* = *j* and 0 otherwise.

#### Quantifying of survival and growth conditions influence

To investigate the influence of complex combination of extracellular conditions on yeast growth kinetic we used the numeric calculation of the OD integral and the O2 integral. The integral was defined as the area under the growth curve or oxygen saturation curve:

$$\text{OD integral}(\text{OD},\text{ t})=\sum_{i=1}^{n}\left(\frac{1}{2}*({\text{OD}}_{i}+{\text{OD}}_{i-1})*({\text{t}}_{i}-{\text{t}}_{i-1})\right)$$

where *n* is the total number of discrete time points in the measurement, and in the case of O2 integral the oxygen saturation of medium was used instead the OD. By using non-parametric GRPR we defined multidimensional model of the OD integral and O2 integral dependence on quantitative influence of multiple extracellular factors. From the integral dependence on acetic acid concentration we defined the EC50 for the growth or mitochondria inhibition as the amount of acetic acid needed for 50% of growth or mitochondria respiratory inhibition:

$$EC_{50}(\text{OD integral}):=\text{c}(\text{acetic acid})$$

with

$$\text{OD integral}(\text{c}(\text{acetic acid}))=\frac{\text{max}[\text{OD integral}]-\text{min}[\text{OD integral}]}{2}$$

where in the case of EC50 for mitochondria inhibition the O2 integral was used.

To estimate relative growth or mitochondria viability we calculated the area below curves that represent OD or O2 integral (calculated from GRPR) dependence on acetic acid concentration and defined these areas as OD toxicity or O2 toxicity integrals. These integrals were further normalized to the highest values for each investigated strain and the resulted normalized parameters were defined as relative growth viability or relative mitochondria viability:

$$\text{viability}(\text{OD integral},\text{ c}(\text{acetic acid}))=\sum_{i=1}^{n}\left(\frac{1}{2}*({\text{OD integral}}_{i}+{\text{OD integral}}_{i-1})*(\text{c}{\left(\text{acetic acid}\right)}_{i}-\text{c}{\left(\text{acetic acid}\right)}_{i-1})\right)$$

where *n* is the total number of grid points in the multidimensional interpolation space, and in the case of O2 integral the oxygen saturation of medium was used instead the OD.

## Results

### Dependence of acetic acid toxicity on the extracellular pH conditions

To study dependence of acetic acid toxicity on the extracellular pH and glucose concentration we performed an automated multifactorial experiment in which the multidimensional experimental space is generated by an algorithm that sequentially adds parameter combinations maximizing the difference to data points already in the experimental design set. The resulting space filling experimental design covers the entire feasible parameter space evenly. By using a log-scaled axis for glucose concentration, the data points in our experimental design provide a dense coverage of the lower concentration range. The calculation of growth kinetic is performed by piece-wise linear regression of log (OD) and the maximal growth rate and maximal biomass yield are determined for each measured set of conditions. The obtained results are used to develop a RSM of growth dependence on extracellular glucose concentration and pH condition by GRPR (see Materials and Methods).

**Figure 1:**
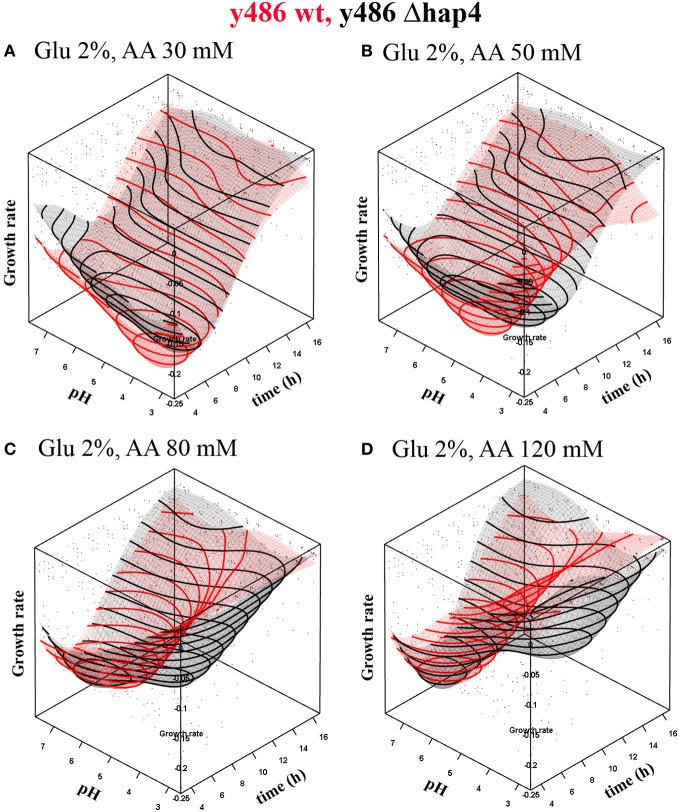
**Dependence of growth rate on pH conditions in YPD medium upon treatment with (A) 30 mM or (B) 50 mM, (C) 80 mM and (D) 120 mM acetic acid and constant 2% glucose concentration.** The graphs represent interpolated response surface models of growth rates (μ) over time in dependence on pH conditions for wild-type (red surface) and hap4 mutant cells (black surface). Note: inverted growth scale; the best growth is in the valley. Individual data point in the graph represent single measurements that were base for the interpolation. As the interpolation surface presented in the graph was based on the standard glucose concentration, data points for other glucose concentration conditions lay outside the interpolated surface.

RSMs of cell growth dynamics confirmed a strong shift of optimal pH conditions from low pH values toward higher ones in presence of increasing acetic acid concentrations (Figure 1). Lack of Hap4p resulted in a slightly reduced growth rate (Figure 1A) and significantly reduced biomass yield (data not shown). Although, the growth of yeast cells under high glucose concentration does not require mitochondrial activity, our results indicate that functional mitochondria and the transcriptional complex Hap 2-3-4-5 are indispensable for optimal growth rates and biomass production. Upon treatment with acetic acid survival is facilitated in conditions that favor the dissociation of acetic acid, e.g., higher pH environment. However, dissociation to acetate anion and hydrogen ion leads to acidification of the extracellular milieu. If the buffering capacity of the medium (50 mM citrate/phosphate buffer) is exceeded by high acetic acid concentrations a considerable amount of acetic acid will be present in undissociated form and able to enter the cell. Therefore, the treatment with very high concentrations of acetic acid will cause significant growth reduction even in high extracellular pH conditions (Figure 1).

The response of Δ*hap4* mutant to acetic acid treatment was significantly different from the response of wild-type cells. Surprisingly, Δ*hap4* mutant cells could survive in much lower extracellular pH conditions (Figure 1). The maximal growth rate of the Δ*hap4* mutant is reached at a later time point of cultivation if cells are grown at lower pH. In contrast, wild-type yeast showed a complete growth inhibition under these conditions. At higher acetic acid concentration (120 mM; Figure 1D) growth rate was also reduced in Δ*hap4* cells.

**Figure 2:**
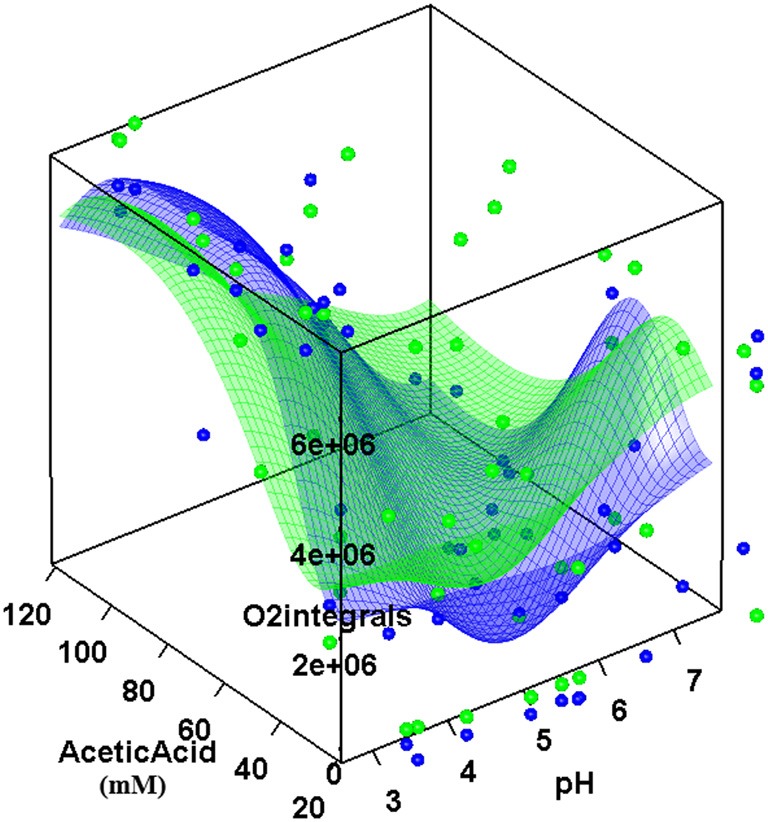
**Respiratory activity dependence on acetic acid concentration and extracellular pH.** The graphs represent interpolated response surface models (RSM) of O_2_ integrals, defined as the area under the oxygen saturation curve, in dependence on pH and acetic acid concentrations for wild-type (blue surface) and hap4 mutant cells (green surface) at a standard glucose concentration in the medium (2%). Note: high integral represents low oxygen consumption and respiratory inhibition; the best respiratory activity is in the valley. Individual data points in the graph represent single measurements that were the basis for the interpolation. The interpolation surface presented in the graph is based on one glucose concentration, and the data points for other glucose concentrations lay outside the interpolated surface.

Presence of acetic acid in growth medium appeared to be toxic for the mitochondria respiratory activity as well (Figure 2). Based on the measurements of oxygen consumption of cells grown in various combinations of extracellular conditions (see Materials and Methods) we developed a RSM of respiratory activity in dependence of acetic acid, glucose concentration, and extracellular pH. The O_2_ integral was defined as the area under the oxygen saturation curve over time during the experiment. These values were used for computing the RSM of the acetic acid inhibition of respiratory activity. The inhibition of respiration was clearly concentration and pH dependent (Figure 2).

**Figure 3:**
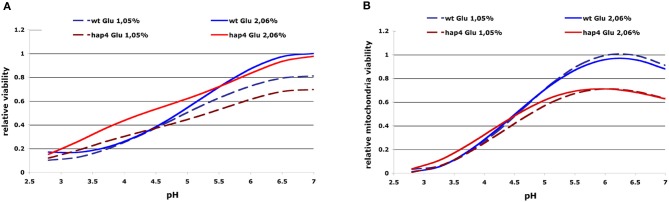
**2D presentation of calculated “relative growth viability” (A) and “relative mitochondrial viability” (B) upon acetic acid treatments in dependence on extracellular pH and glucose concentration.** The curves represent the OD/O_2_-integral dependence on acetic acid concentration calculated from RSM for the whole range of pH values (x-axis) and were normalized to the highest values for each investigated strain. Results for selected glucose concentrations and strains are presented as indicated. The resulting normalized values were defined as “relative growth viability” and “relative mitochondria viability” (see Materials and Methods). Sensitivity to acetic acid is clearly reduced in the hap4 mutant at lower pH, while impaired mitochondrial activity of the Δ*hap4* mutant can be seen at higher pH.

The ability of acetic acid to inhibit cell growth or mitochondria activity was characterized as “relative growth viability” or “relative mitochondria viability” by using non-parametric GRPR we defined multidimensional RSM of the OD-integral and O_2_-integral dependence on multiple extracellular factors (see Materials and Methods). From these models, we calculated the area below curves representing OD- or O_2_-integral dependence on acetic acid concentration calculated from RSM for various pH and glucose concentrations in the medium as OD toxicity or O_2_ toxicity integrals. These integrals were further normalized to the highest values of each strain investigated to obtain normalized values that we defined as “relative growth viability” or “relative mitochondria viability.” With this computational processing we obtained numerical values describing the cellular sensitivity to the complex combination of environmental factors that can be presented in 2D graphs with one variable. Our results clearly show the highest toxicity of acetic acid (growth and respiration) in low pH conditions and lower sensitivity of the Δ*hap4* mutant (Figure 3). At higher pH conditions the Δ*hap4* mutant exhibited reduced mitochondrial respiratory activity. Interestingly, inhibition of respiration by acetic acid was comparable between wild-type and Δ*hap4* mutant suggesting that further metabolic alterations, differently regulated in those strains, are responsible for the increased resistance of the mutant strain to acetic acid (Figure 3B).

### Increased glucose concentration protects cells against acetic acid toxicity

**Figure 4:**
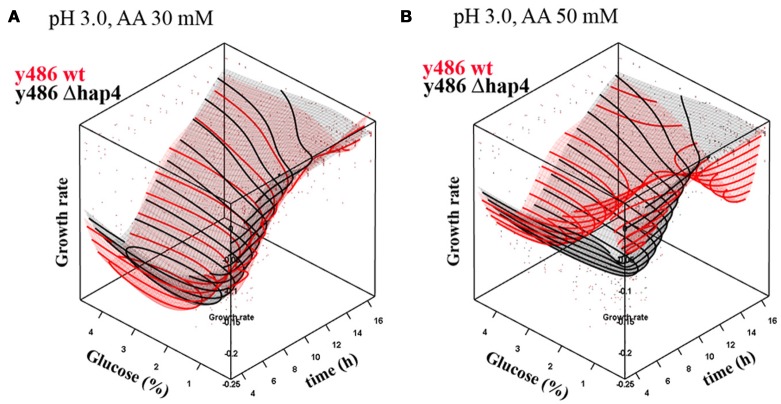
**Dependence of growth rate on glucose concentration in YPD medium upon treatment with (A) 30 mM or (B) 50 mM acetic acid (AA) at pH 3.0.** The graphs represent response surface models of growth rates (μ) over time in dependence on glucose concentration for wild-type (red surface) and hap4 mutant cells (black surface). Note: inverted growth scale; the best growth is in the valley. Individual data points in the graph represent single measurements that were used for the interpolation. The interpolation surface presented in the graph is based on one pH (pH 3.0), and data points for the other pH conditions lay outside the interpolated surface.

Growth rate of yeast cells at pH 3.0, in the presence of very low, non-toxic concentrations (30 mM) of acetic acid, progressively increased over time, reaching a maximum after about 5 h of cultivation (Figure 4A). After this time point growth rate was decreasing as nutrient supply was depleted and metabolic by-products accumulated. Increasing glucose concentration (up to 2%) had a stimulating effect on the growth rate. The optimum glucose concentration under these conditions was between 2% and 4%. Both, wild-type and Δ*hap4* mutant, showed a similar growth dependence on glucose in the medium. Interestingly, when acetic acid concentration was increased to 50 mM (Figure 4B), the optimum glucose concentration also increased, with the optimum being above 3.5%. In Δ*hap4* cells, however, acetic acid did not trigger a shift in optimal glucose levels in the medium, although, the toxic influence of acetic acid, observed as decreased growth rate and delayed time when maximal growth rate is reached, was similar in both strains.

**Figure 5:**
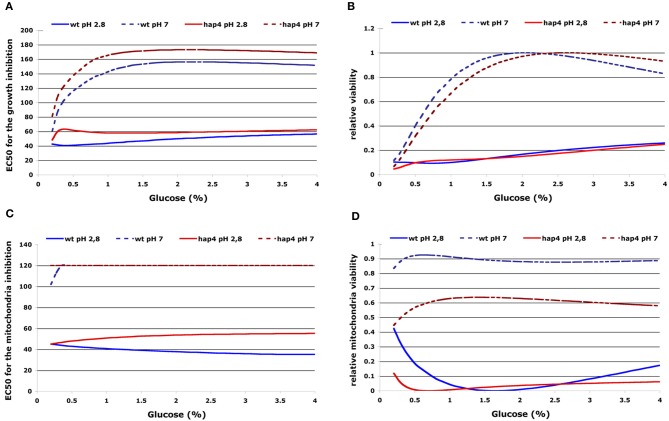
**2D presentation of calculated EC50 for growth inhibition (A) or inhibition of respiration (B) and “relative growth viability” (C) or “relative mitochondria viability” (D) upon acetic acid treatment in dependence on glucose concentration and extracellular pH.** The EC50 for growth or mitochondria inhibition was defined by the integral dependence on acetic acid concentration as the amount of acetic acid needed for 50% of growth or mitochondria respiratory inhibition. “Relative growth viability” or “relative mitochondria viability” were defined as the normalized values that represent OD/O_2_-integral dependence on acetic acid concentration (see Materials and Methods). Results for selected pH values and strains are presented as indicated.

To quantify the protective effect of increased extracellular glucose concentrations on the cellular sensitivity to acetic acid we used the interpolated RSM of the OD integral and O_2_ integral to visualize the influence of multiple extracellular factors. From the integral of the dependence on acetic acid concentration we defined the specific EC50 as the amount of acetic acid needed for 50% of growth or mitochondria respiratory inhibition. As mentioned before, we also calculated the area below curves that represent OD or O_2_ integral dependence on acetic acid concentration calculated from RSM for various pH and glucose concentrations in the medium and defined these areas as OD toxicity or O_2_ toxicity integrals. As before, results were normalized to the highest values for each investigated strain and the resulting normalized parameters were again defined as “relative growth viability” or “relative mitochondria viability.” The results show that increasing extracellular glucose concentrations resulted in an increased EC50 for growth inhibition and inhibition of mitochondrial respiration in both investigated strains (Figures 5A,C). The data also indicate a steady increase of EC 50 values for both, growth and mitochondria inhibition, with increased extracellular pH (Figures 5A,C). These results were also reflected in an increase of “relative growth viability” at high extracellular glucose or pH conditions (Figure 5B). However, “relative mitochondria viability” was decreased at elevated glucose concentrations in the medium as a result of decreased aerobic and increased fermentative metabolism (Figure 5D).

### Effect of acetic acid on glucose uptake and carbohydrate storage

Glucose-repressed cells are impermeable to the anion (Cássio et al., 1987) and only the undissociated form of acetic acid is able to enter the cells by simple diffusion. Acetic acid affects the transport of glucose by acting on the transport proteins as uncoupler dissipating ΔpH and membrane potential (Sousa et al., 1995). Our results show that acetic acid significantly inhibited mitochondria respiration (Figure 2). The inhibitory effect on mitochondria can be explained as a result of respiratory chain uncoupling and an impinging effect on the ΔΨ.

**Figure 6:**
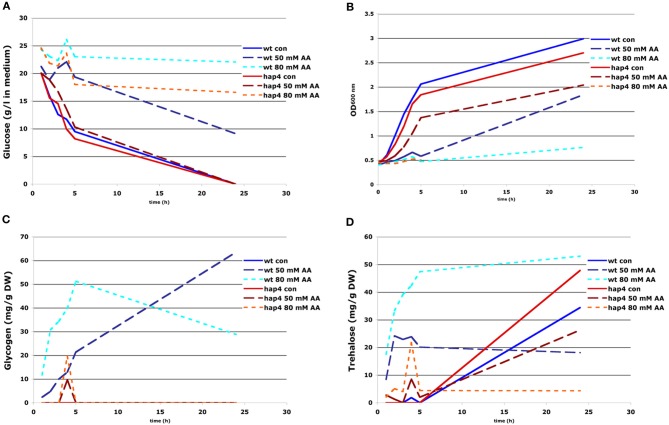
**Effect of acetic acid treatment on cell growth, glucose consumption, and accumulation of glycogen and trehalose.** FF18984 wild-type and hap4 mutant cells were cultured to mid-log phase (OD_600_ = 0.6–1.0) in full (YPD) medium either mock- (control) or acetic acid-treated (AA) with concentration of 30, 50, and 80 mM of AA. Glucose content in the medium **(A)**, optical density of the culture (OD_600_) **(B)**, and kinetics of glycogen **(C)** and trehalose **(D)** accumulation were monitored during 26 h of cultivation.

In order to test whether glucose consumption directly depends on the concentration of acetic acid we performed time-course measurements of glucose concentration in presence of increasing concentrations of acetic acid. The results confirmed an acetic acid concentration-dependent inhibition of glucose uptake (Figure 6A). Surprisingly, the inhibitory effect of acetic acid observed was much lower in the Δhap4 mutant. Although reduced, glucose consumption was still detected in Δhap4 mutant treated with 50 mM acetic acid. In contrast, wild-type cells treated with the same acetic acid concentration completely ceased glucose consumption already after 2 h of treatment (Figure 6A). Moreover, the inhibition of growth by acetic acid was reciprocally proportional to the inhibition of glucose consumption (Figure 6B). Thus, sustained glucose uptake in Δhap4 mutant supported cell growth even in the presence of 50 mM acetic acid.

Respiratory deficient cells exhibit high level of glycogen and trehalose accumulation (Enjalbert et al., 2000; Kitanovic et al., 2009). In general, various types of stress, like nutrient starvation, heat-shock, or oxidative stress, induce futile cycling of storage carbohydrates, trehalose and glycogen, meaning that both biosynthesis and biodegradation pathways are activated almost to the same extend (Parrou and Francois, 1997). Glycogen and preferentially trehalose, serve as a fuel reserve that enable yeast cells to survive starvation. Upon return to favorable conditions or upon defeating stress conditions these storage carbohydrates can be quickly mobilized to help fuel growth (Shi et al., 2010). However, in conditions where glucose uptake is inhibited by acetic acid, increased synthesis of glycogen and trehalose would create significant energy deficiency and dissipation of important metabolic intermediates that could be used in catabolic reactions to sustain sufficient levels of ATP within cells. To test this hypothesis, we measured glycogen and trehalose accumulation upon treatment of wild-type and Δhap4 mutant with increasing acetic acid concentrations. The experiments showed that acetic acid treatment caused significant accumulation of both, glycogen and trehalose only in wild-type cells (Figures 6C,D). In the Δhap4 mutant we detected increased levels of these storage carbohydrates only at the onset of glucose exhaustion, the conditions which normally facilitate their accumulation. A short, transient increase of trehalose and glycogen levels followed by their fast mobilization was observed in Δhap4 mutant only after treatment with very high acetic acid concentrations (80 mM; Figures 6C,D).

### Intracellular acidification and glycolytic flux upon acetic acid treatment

Yeast cells are unable to maintain a stable pH gradient across the plasma membrane on starvation (Dechant et al., 2010). It was shown earlier that reduced glycolytic flux upon starvation directly results in significant acidification of the cytosol (Dechant et al., 2010) as a consequence of ATP depletion. For ATPase to accomplish its function of regulating internal pH (pHin), metabolic activity and sustainable level of ATP is required. Therefore, it is to be expected that increased glucose concentration in the medium could help to keep sufficient ATP level for ATPase function and prevent intracellular acidification.

**Figure 7:**
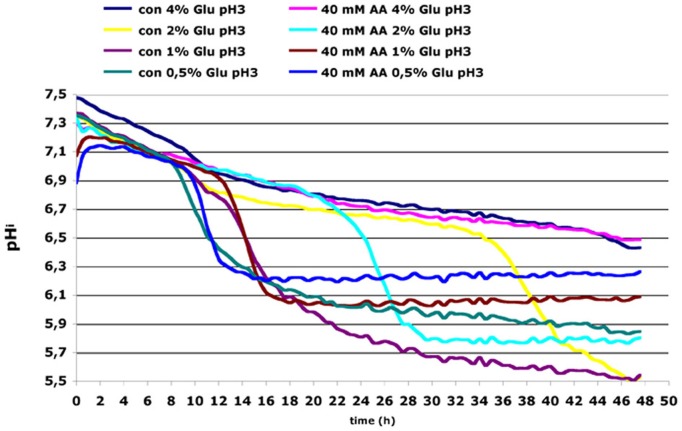
**Wild-type (wt) yeast cells transformed with pHluorin containing reporter plasmid were incubated in a low fluorescent F1 medium at pH 3 containing increasing concentrations of glucose (0.5, 1, 2, and 4% Glu).** Equal amounts of cells were plated in each well of 96-well microtiter plate with round bottom. Cells were either mock-treated (con) or treated with 40 mM acetic acid. The kinetic of the fluorescence signal (Ex/Em pairs 390/510 and 480/510) was monitored in a plate reader. The intracellular pH was calculated based on a calibration curve that was made with the same cells incubated in phosphate/citrate buffer adjusted to pH between 5 and 8 in presence of 0.16 % digitonin.

To investigate alterations of intracellular pH upon acetic acid treatment we performed *in vivo* monitoring of cytosolic pH using ratiometric measurements with pHluorin that exhibits pH-dependent dual excitation peaks at 395 and 475 nm (Braun et al., 2010). Cells containing reporter plasmid were grown in minimal SD medium with different glucose concentrations in presence or absence of 40 mM acetic acid. In control culture grown in extracellular pH 3.0 and 2% glucose a fast drop of intracellular pH was observed in the late stationary phase (Figure 7). Acetic acid treatment under these conditions resulted in much earlier cytosolic acidification already after 20 h of treatment probably as a consequence of ATP depletion following inhibition of glucose uptake (as shown in Figure 6A). Cultivation of cells in 4% glucose medium could prevent intracellular acidification in both control and acetic acid treated cells. In contrast, decrease of glucose concentration in medium resulted in rapid cytosolic acidification at a much earlier time point of cultivation independently of acetic acid. These results confirmed that maintenance of intracellular pH depends on glucose signaling. In conditions of glucose exhaustion in the medium or inhibition of glucose uptake by acetic acid, the intracellular ATP pool becomes limited and—as a consequence—disturbs the pH balance, significantly reducing cytosolic pH.

Dechant et al. (2010) showed that changes in the ATP level directly impinge on cytosolic pH, which acts as a second messenger in response to glucose. A similar mechanism of glucose sensing was described in pancreatic β-cells, in which increasing ATP concentrations mediate glucose sensing through inactivation of ATP-dependent K^+^ channels (MacDonald and Wheeler, 2003). Our results show that trehalose and glycogen were synthesized in acetic acid treated cells even in absence of glucose uptake. Together with the observation of Almeida et al. (2009) that amino acids pools are significantly reduced as a consequence of acetic acid treatment, it can be concluded that acetic acid treatments should lead to gluconeogenesis and consumption of glycolytic and tricarboxilic acid pathway (TCA) intermediates in favor of storage carbohydrate synthesis.

**Figure 8:**
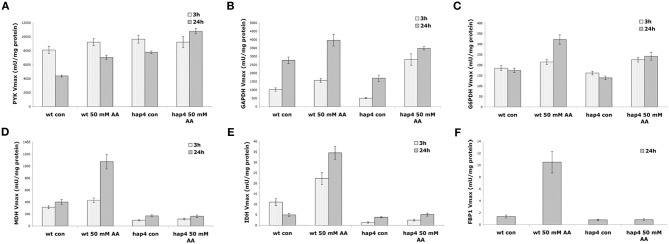
**Effect of acetic acid treatment on the activity of metabolic enzymes.** Cultures of yeast wild-type (wt) and Δhap4 mutant cells growing exponentially in full (YPD) medium at pH 3 were divided into two batches, one of which was mock-treated (con), the other challenged with 50 mM acetic acid (AA). At the indicated times of incubation aliquots were withdrawn and **(A)** pyruvate kinase (PYK), **(B)** glyceraldehyde-3-phosphate dehydrogenase (GAPDH), **(C)** glucose-6-phosphate dehydrogenase (G6PDH), **(D)** malate dehydrogenase (MDH), **(E)** isocitrate dehydrogenase (IDH), and **(F)** fructose 1,6 bisphosphatase (FBP1) activities were measured from the cellular protein extract. The values are given in mU/mg protein ± SE (U = μmol/min).

To test this hypothesis we analyzed the activity of glycolytic and TCA pathway enzymes upon treatment with 50 mM acetic acid in wild-type and the Δ hap4 mutant. The results obtained (Figures 8A–C) show a significant increase in the activity of the glycolytic enzymes PYK, GAPDH, and G6PDH activity in both wild-type and Δhap4 mutant cells. Higher concentration of acetic acid (from 80 mM) significantly inhibited the activity of all three enzymes (data not shown). The activity of the TCA pathway enzymes MDH, and IDH, and the key enzyme in gluconeogenesis, FBP1, however, was elevated only in wild-type cells. The Δhap4 mutant showed significantly reduced activity of those enzymes in both control and acetic acid treated cells.

The yeast *Saccharomyces cerevisiae* adapts to glucose exhaustion through induced transcription of genes involved in various cellular processes, including gluconeogenesis, the glyoxylate cycle, the tricarboxylic acid (TCA) cycle, respiration, β-oxidation, and utilization or transport of alternative sugars. Enzymes of gluconeogenesis and the glyoxylate cycle are indispensable for growth on non-fermentable carbon sources, such as, ethanol, lactate, or glycerol (Schüller, 2003). Hap4p is a subunit of the Hap2/3/4/5 transcriptional complex, which is involved in the transcriptional regulation of TCA cycle genes, glyoxylate cycle genes and stress response genes (Raghevendran et al., 2006). Hap4p overexpression results in increased growth rates and biomass formation and prolonged life span (Lin et al., 2002). Therefore, decreased toxicity of acetic acid in Δ hap4 mutant could be explained by lower inhibition of glucose uptake and, in parallel, decreased activity of gluconeogenesis, TCA cycle, and synthesis of storage carbohydrates. This would result is increased ATP levels that could support the function of membrane ATPases in maintaining intracellular pH homeostasis and prevent amino acids starvation.

## Discussion

The accumulation of acetic acid in stationary phase budding yeast cultures is considered to be the primary mechanism of chronological aging in yeast and recent results suggest that the mechanism of acetic acid toxicity in yeast could be related to the induction of growth signaling pathways and oxidative stress (Burhans and Weinberger, 2009; Burtner et al., 2009). Recent publications showed that buffering medium could inhibit the age-dependent accumulation of reactive oxygen species preferentially superoxide anions that is produced by dysfunctional mitochondria (Burhans and Weinberger, 2009; Pan et al., 2011). The accumulation of acetic acid in stationary phase induces oxidative stress, a factor previously implicated in chronological aging of yeast and aging in other organisms as well. Interestingly, the accumulation of acetic acid in stationary phase cultures inhibits growth arrest of cells in G1 and is preferentially toxic to cells that fail to undergo a G1 arrest (Burhans and Weinberger, 2009).

We used a multifactorial experimental design to investigate the impact of acetic acid on cellular growth kinetics in dependence on glucose concentration as well as extracellular pH, covering the whole range of combinations in experimental conditions. Raising the extracellular pH clearly reduced the toxic influence of acetic acid. The accumulation of undissociated acids within the cell is a function of Δ pH and glucose concentration in the medium (Thomas et al., 2002). By raising the pH to a value higher than the pKa of the acid, the concentration of undissociated acid is reduced for a given amount of total acid, placing less stress on cells. Raising the extracellular glucose concentration results in increased intracellular ATP supporting the activity of ATPases (Thomas et al., 2002). Both conditions result in a lower waste of energy for maintenance of the pHin in the range optimal for growth. The outcome is a decreased inhibitory effect of acetic acid on yeast growth and metabolism.

The protecting effect of high glucose concentrations observed in our experiments could be explained by several mechanisms. Acetic acid can enter the cells only in its undissociated, uncharged form. The charged acetate anion is generally considered as non-toxic (Piper et al., 2001). A higher pH on the cytosolic side of the membrane can cause a substantial fraction of this acid to dissociate to the anion, a form which is relatively membrane-impermeable and that therefore will accumulate inside the cell resulting in intracellular acidification (Piper et al., 2001).

In budding yeast and many other fungi, intracellular acidification activates highly conserved Ras2 and cAMP-dependent signaling pathways that respond to glucose (Thevelein and de Winde, 1999). Thus, despite the oxidative stress, inhibition of glycolysis, induction of gluconeogenesis and synthesis of storage carbohydrates, acetic acid treated cells are continuously subjected to growth signals that promote entry into S phase. Constitutive activation of the Ras-cAMP-PKA pathway would also result in a PKA-dependent loss of mitochondrial function (Gourlay and Ayscough, 2006), which requires *HAP4* transcriptional regulation and accumulation of damaged, high ROS producing mitochondria (Leadsham and Gourlay, 2010). The result is a conflicting situation. On one side, inhibition of glucose uptake and mitochondria function by acetic acids will result in significant ATP depletion and disturbed pH homeostasis. Available nutrients are then redirected toward synthesis of storage carbohydrates causing insufficient synthesis of dNTPs and inefficient DNA replication. On the other side, Ras-cAMP-PKA activation will stimulate growth. Together, intracellular acid accumulation seems to trigger an inappropriate growth signal and replication stress, which leads to cell death. Feeding with high glucose concentrations can, therefore, prevent the energetic collapse in mitochondria impaired cells where the glycolytic flux is reduced because of low pH. This also fits with the observation that neutralizing buffering of yeast media could extend chronological life span in yeast cells, implicating that other mechanism than just simple acidification of the environment are involved in acetic acid-induced metabolic alterations and apoptosis induction (Pan et al., 2011).

Reduced sensitivity of the Δhap4 mutant to acetic acid may be explained by impaired expressions of gluconeogenic, glyoxylate, and TCA cycle enzymes, which are regulated by the Hap 2-3-4-5 complex (Figure 8). The low gluconeogenic activity in the Δ hap4 mutant will prevent the synthesis of storage carbohydrates, trehalose and glycogen, in conditions where glucose uptake is inhibited by acetic acid. Lin et al. (2002) showed that aged cells respond to glucose-deprivation by shifting the metabolism away from glycolysis toward gluconeogenesis and energy storage. In acetic acid induced aging, increased gluconeogenesis and trehalose/glycogen synthesis pathways will compete with amino acids synthesis pathways for the same glycolytic and TCA intermediates. In consequence, this will result in a condition of amino acids starvation and ATP depletion. In contrast, in the Δhap4 mutant the limited available glucose in the cells can be fully used for sustaining the ATP pool and pHin homeostasis and significantly decrease acetic acid toxicity.

Our results clearly show the interdependence between an important metabolic by-product of yeast fermentation, acetic acid, and the efficiency of the cellular metabolism as well as aging of cells. In addition to providing some new insight into the role of extracellular conditions and availability of nutrients on glucose metabolism, respiration, cellular proliferation and aging, which play a central role in different diseases, our results also suggest controlled stress conditions as a means to increase fermentation efficiency. Given the urgent need to optimize the production of fuel ethanol from cellulosic biomass as a more environmental-friendly fossil fuel alternative, our results suggest that investigating the role of metabolic by products and other stress conditions on fermentation could still lead to further optimization.

## Author contributions

Ana Kitanovic and Stefan Wölfl planed and designed the project. Felix Bonowski conceived and designed the computational framework, Ana Kitanovic, Florian Heigwer, Igor Kitanovic, and Christin Ungewiss designed and performed experiments, Ana Kitanovic, Florian Heigwer, Peter Ruoff, and Stefan Wölfl modeled experimental results, Ana Kitanovic, Igor Kitanovic, and Stefan Wölfl wrote the manuscript.

### Conflict of interest statement

The authors declare that the research was conducted in the absence of any commercial or financial relationships that could be construed as a potential conflict of interest.
